# Pleckstrin-2 promotes the progression of colorectal cancer via YTHDF2-mediated TYMS mRNA stability

**DOI:** 10.1007/s00018-025-05782-x

**Published:** 2025-07-19

**Authors:** Qian Zhou, Yanxia Li, Xiaomei Li, Shujing Zhang, Ying Wang, Zhuoran Li, Xia Wang, Yuan Li, Jingxin Li, Chunhua Lu, Yuemao Shen, Baobing Zhao

**Affiliations:** 1 Key Lab of Chemical Biology (MOE), School of Pharmaceutical Sciences, Cheeloo College of Medicine, Shandong University, Jinan, 250012 Shandong China; 2 NMPA Key Laboratory for Technology Research and Evaluation of Drug Products, School of Pharmaceutical Sciences, Cheeloo College of Medicine, Shandong University, Jinan, 250012 Shandong China; 3 Department of Pharmacology, School of Pharmaceutical Sciences, Cheeloo College of Medicine, Shandong University, NO. 44 Wenhua Road, Jinan, Shandong 250012 P.R. China; 4 Department of Physiology, School of Basic Medical Sciences, Cheeloo College of Medicine, Shandong University, Jinan, 250012 Shandong China

**Keywords:** Pleckstrin-2, YTHDF2, TYMS, Colorectal cancer, M^6^A modification

## Abstract

High expression of nucleotide synthetic enzyme thymidylate synthase (TYMS) is responsible for the resistance to fluorouracil (FU) treatment and worse survival in colorectal cancer (CRC). Herein, we revealed that pleckstrin-2 (PLEK2) cooperated with YTHDF2 to enhance *TYMS* mRNA stability in CRC via an m^6^A dependent manner*.* Silencing of *PLEK2* led to the degradation of *TYMS* mRNA that suppressed DNA replication, which activated p53/p21 signaling and consequent inhibition of CRC cell proliferation via the cellular senescence. Additionally, PLEK2 is also required for CRC cell migration, invasion and stemness-like properties. PLEK2 inhibition is sufficient to ameliorate the progression of AOM/DSS-induced CRC*.* Together, our study identified PLEK2 as a key regulator for the progress of CRC via the regulation of *TYMS* expression, and demonstrated that PLEK2 is a novel therapeutic target for CRC.

**Supplementary Information:**

The online version contains supplementary material available at 10.1007/s00018-025-05782-x.

## Introduction

Single-agent fluorouracil (FU) or its combination with new classes of drugs such as oxaliplatin and irinotecan, has been the main treatment of choice in colorectal cancer (CRC) [1, 2]. A primary mechanism of action of FU is inhibition of the nucleotide synthetic enzyme thymidylate synthase (TYMS), resulting in proliferative inhibition. TYMS is a cytosolic enzyme that catalyzes the reductive methylation of deoxyuridine monophosphate (dUMP) to yield deoxythymidine monophosphate (dTMP), which is required for DNA synthesis and repair [3]. Recent studies have demonstrated that the high expression of TYMS is responsible for the resistance to FU treatment and worse survival in CRC [4–6].

The N (6)-methyladenosine (m^6^A) modification has been identified as one of the post-transcriptional regulatory markers in different types of RNAs, which play important roles for regulating RNA stability, translation, splicing and translocation [7]. Emerging evidence suggests that abnormal m^6^A modification is involved in the progression, metastasis, drug resistance and prognosis of malignant tumors [8, 9], including CRC tumorigenesis [10]. m^6^A modification is installed by the m^6^A methyltransferases (writers), such as METTL3/14, and removed by the demethylases (erasers). Proteins containing the YT521-B homology (YTH) domain, including YTHDF1, 2, and 3 in mammals, have been identified as m^6^A readers recognizing and binding to the m^6^A-modified RNAs [11–13]. YTHDF1 enhances translation of its targets by interacting with initiation factors and facilitating ribosome loading [14]. Although YTHDF2 has been reported to facilitate protein translation via the binding to the 5’UTR of target mRNAs under stress conditions [15], it mainly accelerates the decay of its mRNA targets via recruiting RNA decay machinery factors on the 3’UTR of mRNA [11, 16]. YTHDF3 serves as a hub of YTHDF1 and 2 to facilitate translation and decay of m^6^A-modified RNA [17].

YTHDF2 has been documented as an oncogene in CRC by promoting the decay of targeted mRNAs [18–22]. Surprisingly, in this work, we demonstrated that YTHDF2 promotes *TYMS* mRNA stability via cooperation with Pleckstrin-2 (PLEK2) in an m^6^A-dependent manner in CRC cells. PLEK2 is a member of Pleckstrin family widely expressed in multiple tissues [23]. As a membrane protein and paralog of PLEK1, PLEK2 is involved in the formation of large lamellipodia and peripheral ruffle of cells [23]. Recent studies demonstrated that PLEK2 is highly expressed and associated with worse prognosis and overall survival in various cancers [23, 24]. The mechanisms of PLEK2 in tumorigenesis is tissue specific, as PLEK2 mediates proliferation and metastasis in non-small cell lung cancer via SHIP2/PI3K/AKT and bromodomain containing protein 4 expression [25, 26]. PLEK2 was also reported to promote tumorigenesis and metastasis by activating the PI3K/AKT, EGFR and TGF-β signaling [27–29], as well as a downstream substrate of the APC/β-catenin signaling [30]. Herein, our studies suggest that PLEK2 is an attractive target for the treatment of cancers via the regulation of *TYMS* expression.

## Results

### PLEK2 was upregulated in CRC

**Fig. 1:**
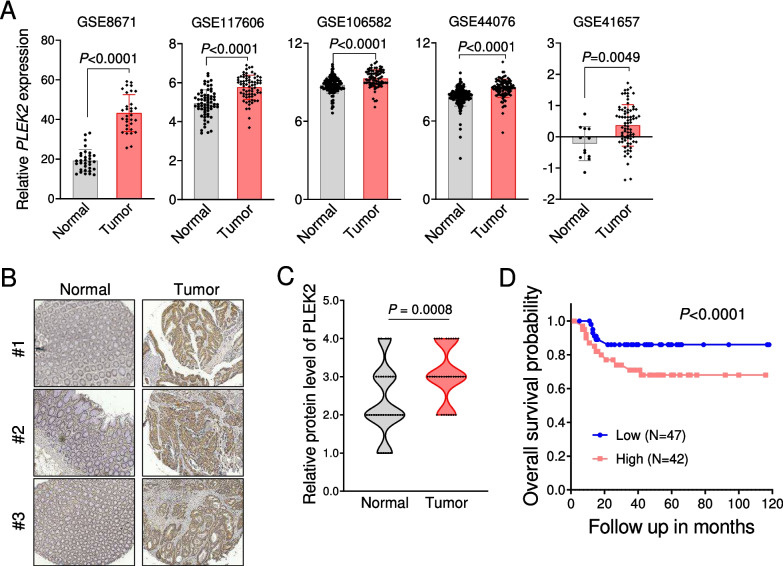
PLEK2 is a potential unfavorable prognostic marker in CRC. (**A**) RNA-seq expression profiles of *PLEK2* in colorectal cancer and paired para cancerous tissues from public databases (GSE8671, GSE117606, GSE106582, GSE44076 and GSE41657). (**B**-**C**) IHC analysis of PLEK2 in the tissues from CRC patients’ tissues microarray. Representative images of IHC staining (B) and the statistical analysis (C) is shown. (**D**) Kaplan–Meier plot of overall Survival of CRC patients was stratified by PLEK2 expression level (GSE33114). See also Fig. S1

To explore the potential role of PLEK2 in cancers, we analyzed the mRNA levels of PLEK2 with the published next-generation sequencing data from Oncomine comprising major types of human cancer with respective normal tissues [31]. Notably, PLEK2 is highly expressed in multiple types of cancer including CRC (Fig. S1A). Individual CRC datasets from Gene Expression Omnibus (GEO) databases also showed that PLEK2 was significantly upregulated in primary tumor tissues of CRC compared with adjacent normal tissues (Fig. 1A). In addition, CRC cells exhibited the increased expression of PLEK2 (Fig. S1B). In line with these findings, our immunohistochemical staining revealed that PLEK2 protein levels were also elevated in CRC tissues compared with normal colorectal tissues (Fig. 1B, C). Further analysis of CRC datasets from GEO database showed that patients with high expression of PLEK2 suffered significantly worse overall survival (Fig. 1D). These data demonstrate that PLEK2 is upregulated in CRC and indicate its potential role in the progress of CRC.

### Silencing of PLEK2 inhibited the CRC cell proliferation via the cellular senescence

**Fig. 2:**
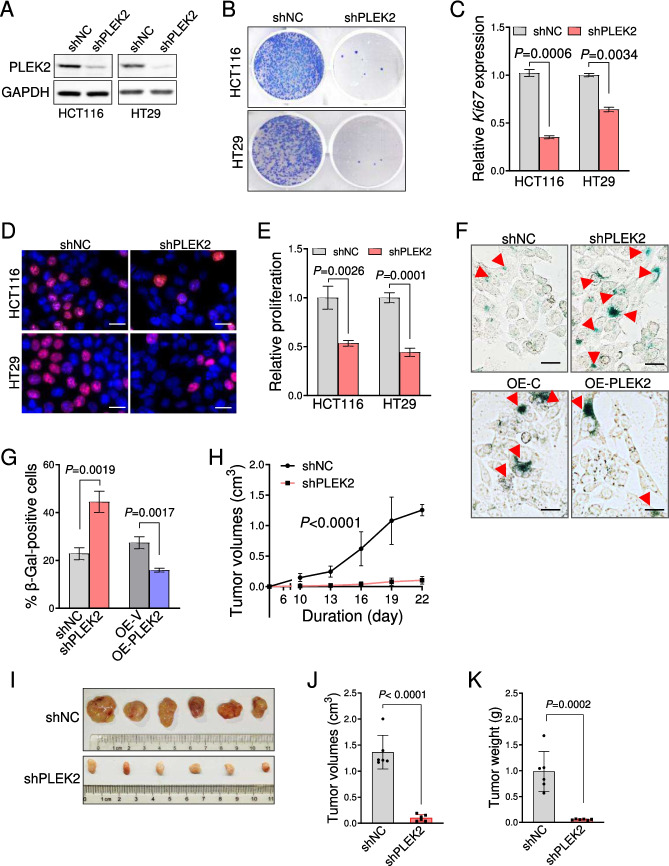
Silencing PLEK2 inhibited the proliferation of CRC cells. (**A**) Immunoblotting analysis of PLEK2 in HCT116 and HT29 cells transduced with retroviruses encoding indicated shRNAs. GAPDH was used as a loading control. shNC represents a non-targeting shRNA. (**B**) Representative images of colony formation assay of HCT116 and HT29 cells transduced with retroviruses encoding indicated shRNAs. shNC represents a non-targeting shRNA. (**C**) Quantitative PCR analysis of *MKI67* mRNA level in HCT116 and HT29 cells transduced with retroviruses encoding indicated shRNAs. shNC represents a non-targeting shRNA. Data were presented as mean ± SD from three independent experiments. (**D**) Representative images of Edu staining in HCT116 and HT29 cells transduced with retroviruses encoding indicated shRNAs. shNC represents a non-targeting shRNA. Scale bars, 20 µm. (**E**) Quantification of EdU positive cells in D. Data were presented as mean ± SD from three independent experiments. (**F**) Representative images of SA-β-gal staining in HCT116 cells transduced with indicated retroviruses treated in the presence of Doxorubicin (0.3 μM) for 24 h. Red arrows indicated senescent cells. OE-C represents overexpression of blank vector, and shNC represents a non-targeting shRNA. Scale bars, 25 µm. (**G**) Quantification of SA-β-gal positive cells in F. Data were presented as mean ± SD from three independent experiments. (**H**) HCT116 cells transduced with retroviruses encoding indicated shRNAs, were subcutaneously transplanted into nude mice. Tumor volumes were measured every 3 days. Data were presented as mean ± SD. *N* = 6 mice for each group. *P* value was determined by two-way ANOVA. shNC represents a non-targeting shRNA. (**I**) Representative images of tumors from H. (**J-K**) Quantification of tumor sizes from the mice as in H on day 22. Each dot represents one mouse. Data were presented as mean ± SD. See also Fig. S2

To determine the role of PLEK2 in CRC, we performed cell proliferation assays in CRC cells HCT116 and HT29 transduced with lentivirus encoding *PLEK2* shRNA (Fig. 2A). *PLEK2* knockdown led to the substantial inhibition of two CRC cells proliferation (Fig. S2A). This was further confirmed by the reduced colony formation in HCT116 and HT29 cells with *PLEK2* silencing (Fig. 2B). To clarify the effects of PLEK2 on the CRC cell growth, we examined the cell cycle and cell viability and found that silencing of *PLEK2* led to a significant increase of cell frequency at G_0_/G_1_ phase but not obvious cell death (Fig. S2B-S2C and date not shown). Moreover, the *MKi67* mRNA level was also reduced after *PLEK2* knockdown (Fig. 2C). EdU assay demonstrated that PLEK2 deficiency impaired the DNA replication in HCT116 and HT29 cells (Fig. 2D, E).

Considering that cell cycle arrest and proliferative inhibition are the typical characteristics of cellular senescence, we analyzed whether *PLEK2* knockdown trigger the cellular senescence in CRC cells. Senescence-associated-β-galactosidase (SA-β-gal, a biomarker of senescence) staining showed that the percentage and strength of SA-β-gal-positive cells were remarkably increased in HCT116 cells with *PLEK2* knockdown (Fig. 2F, G). On the contrary, *PLEK2* overexpression provided an obvious protective benefit for HCT116 cells against the doxorubicin-induced senescence (Fig. 2F, G). Similar findings were also observed in HT29 cells (Fig. S2D-S2E). In addition, *PLEK2* knockdown significantly increased the sensitivity of 5-Fu on HCT116 cells (Fig. S2F).

We next used the xenograft mice model to confirm the role of PLEK2 in CRC cell proliferation *in vivo*. As expected, *PLEK2* knockdown led to the marked reduction in tumor growth of HCT116 cells (Fig. 2H). Correspondingly, tumor volumes and weight in sh*Plek2* group were significantly reduced compared with that of control group (Fig. 2 2I-K and Fig. 2G).

### PLEK2 is required for CRC cell migration, invasion and stemness-like properties

**Fig. 3:**
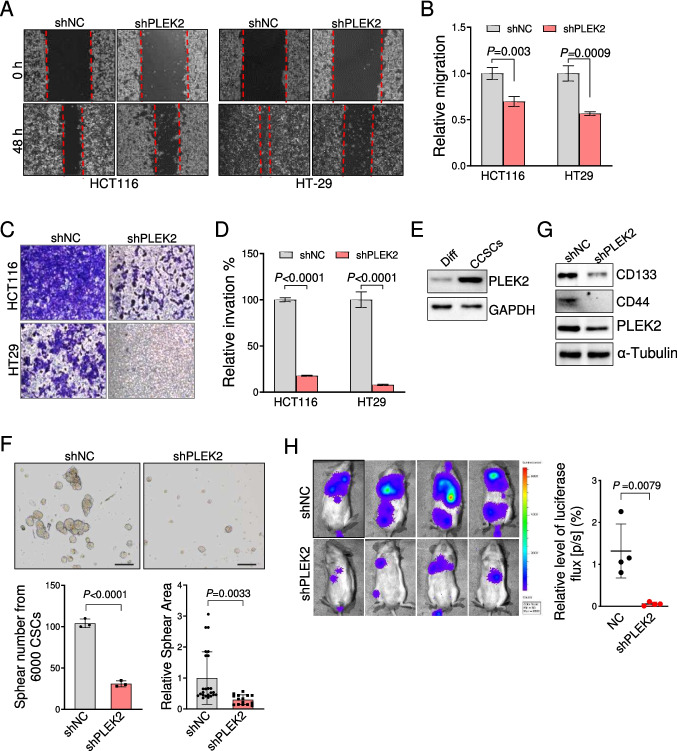
PLEK2 promotes CRC cell migration, invasion and stemness-like properties. (**A**) In vitro wound-healing/scratch assays with HCT116 and HT29 cells transduced with retroviruses encoding indicated shRNAs. shNC represents a non-targeting shRNA. (**B**) Quantitative analysis of cells migration in A. Recovered area with cells were calculated and normalized to the corresponding control group. Data were presented as mean ± SD from three independent experiments. (**C**-**D**) Invasion assays of HCT116 and HT29 cells transduced with retroviruses encoding indicated shRNAs. Representative microscopic fields of invasion cells on the bottom of transwell inserts were shown (C). Quantitation of cell invasion by counting invaded cells from five microscopic fields (D). shNC represents a non-targeting shRNA. (**E**) Immunoblotting analysis of PLEK2 in indicated cells. Diff represents the differentiated colorectal cancer stem cells (CCSCs) generated from CCSCs by culturing in 3% serum medium for 48 h. (**F**) Sphere formation activity of CCSCs transduced with retroviruses encoding indicated shRNAs. Data were presented as mean ± SD from three independent experiments. shNC represents a non-targeting shRNA. Scale bars, 100 µm (**G**) Immunoblotting analysis of stemness-associated proteins in HCT116 cells transduced with retroviruses encoding indicated shRNAs. shNC represents a non-targeting shRNA. (**H**) Representative images of CCSCs metastasis in NXG mice. CCSCs stably expressed luciferase were transduced with retroviruses encoding *PLEK2* shRNAs or shNC (1 × 10^6^/mouse), and then injected into NXG mice via the tail vein. CCSCs metastasis were quantitated using the bioluminescence imaging of luciferase after 7 weeks of injection (right). Each dot represents one mouse. Data were presented as mean ± SD. shNC represents a non-targeting shRNA**.** Data were presented as mean ± SD from three independent experiments. *P* value was determined by two-way ANOVA. See also Fig. S3

To further investigate the functional role of PLEK2 in CRC, we evaluated the effect of *PLEK2* knockdown on the CRC cell metastasis. Wound healing assays demonstrated that *PLEK2* knockdown led to the substantial decrease of the migration in HCT116 and HT29 cells (Fig. 3A, B). Furthermore, we performed the transwell assays and found that PLEK2 deficiency impaired the invasion of these cells (Fig. 3C, D).

CRC stem cells (CCSCs) are a group of cells in tumor tissues that have the characteristics of stem cells such as self-renewal, multi-directional differentiation and tumorigenesis, which account for the higher metastasis and invasion of CRC [32, 33]. Notably, PLEK2 was highly expressed in colorectal CCSCs compared to the differentiated CRC cells (Fig. 3E). Silencing of *PLEK2* significantly reduced the sphear number and size derived from colorectal CCSCs, indicating that *PLEK2* knockdown impaired the proliferation of colorectal CCSCs (Fig. 3F). This was reversely confirmed by the increased sphear number in *PLEK2*-overexpression colorectal CCSCs compared with the control group (Fig. S3A-S3B). Moreover, CCSCs specific markers were also markedly down-regulated after *PLEK2* knockdown, including CD44 and CD133 (Fig. 3G).

Considering that cancer stem cells are responsible for the tumor metastasis, we further evaluated the effect of PLEK2 on the CRC metastasis driven by CCSCs *in vivo*. CCSCs stably expressed luciferase were transduced with retroviruses encoding *PLEK2* shRNA or control shRNAs, and injected into NXG mice via the tail vein (Fig. S3C). We found that *PLEK2* knockdown in CCSCs significantly decreased metastatic potential to kidney, lung and liver in xenograft models after 7 weeks of injection, as demonstrated by decreased number of metastatic nodules (Fig. 3H and Fig. S3D). Histological features of metastatic tumor CCSCs in engraftment organs were characterized by HE staining (Fig. S3E). These results indicated that PLEK2 play an important role in the self-renewal and metastatic capacity of CCSCs.

### *PLEK2* regulated the expression of *TYMS* in CRC cells

**Fig. 4:**
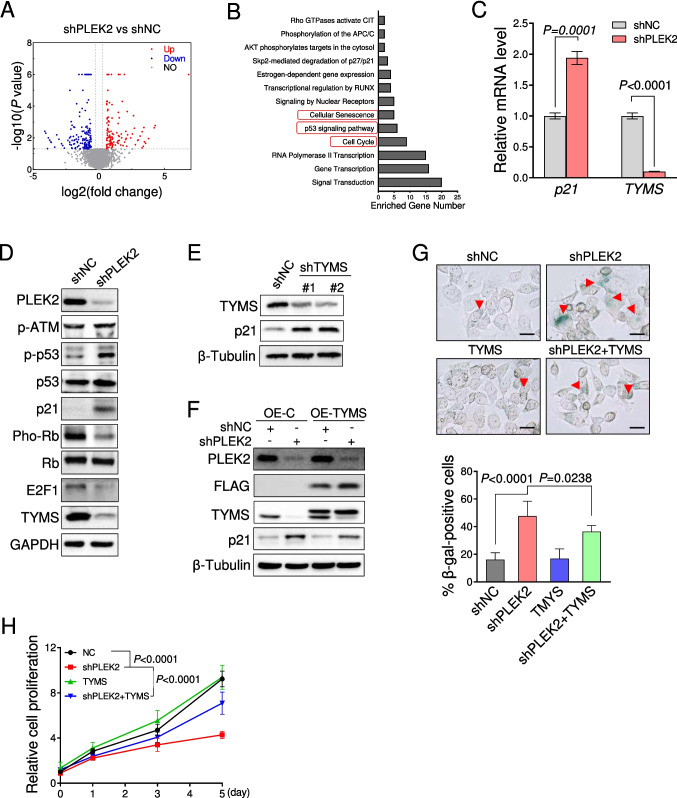
PLEK2 knockdown led to cell senescence via the regulation of TYMS. (**A**) Volcano plot for differentially expressed genes in HCT116 cells transduced with retroviruses encoding indicated shRNAs. shNC represents a non-targeting shRNA. |Fold Change|> 1.5, *P* < 0.05. (**B**) Gene ontology enrichments of differentially expressed genes in A using DAVID bioinformatics database. (**C**) Quantitative PCR analysis of *p21* and *TYMS* mRNA levels in HCT116 and HT29 cells transduced with retroviruses encoding indicated shRNAs. Data were presented as mean ± SD from three independent experiments. shNC represents a non-targeting shRNA. (**D**) Western blotting analysis of indicate proteins in HCT116 cells transduced with retroviruses encoding indicated shRNAs. GAPDH was used as a loading control. shNC represents a non-targeting shRNA. (**E**) Western blotting analysis of indicate proteins in HCT116 cells with retroviruses encoding *TYMS* shRNAs. β-Tubulin was used as the loading control. shNC represents a non-targeting shRNA. (**F**) Immunoblotting analysis of indicated proteins in HCT116 cells with PLEK2 shRNA and the presence of TYMS overexpression. β-Tubulin was used as the loading control. OE-C represents overexpression of blank vector. shNC represents a non-targeting shRNA. (**G**) Representative images and quantification of SA-β-gal staining positive cells in HCT116 cells. Scale bar, 25 μm. (**H**) Quantification of cell proliferation in HCT116 cells as in F. *P* value was determined by two-way ANOVA. See also Fig. S4

To understand the underlying basis of the impaired CRC cell proliferation induced by *PLEK2* deficiency, we performed bulk RNA sequencing of HCT116 cells with or without *PLEK2* knockdown. A total of 306 differentially expressed genes (DEG) were identified (≥ 1.5-fold, *P* < 0.05) (Table S1), including genes related to cell cycle and cell senescence (Fig. 4A, B). In parallel, we also performed the quantitative proteomic analysis and found that p21 and TYMS were the most changed proteins after *PLEK2* knockdown (Fig. S4A). In line with this, *p21* and *TYMS* mRNA levels were significantly altered in HCT116 cells with PLEK2 deficiency (Fig. 4C).

KEGG enrichment analysis revealed that p53 signaling pathway was substantially enriched in the *PLEK2*-shRNA group (Fig. S4B). Indeed, *PLEK2* knockdown led to the increased phosphorylation of ATM and p53 and subsequent p21 expression (Fig. 4D). To determine whether PLEK2 regulated p21 and TYMS expression in a p53-independent manner, we examined the effect of PLEK2 on the HCT116-p53^−/−^ cells, a p53-null originating from HCT116 cells. Notably, p53 deletion largely abolished the upregulation of p21 but not the reduced TYMS induced by *PLEK2* knockdown (Fig. S4C-S4D). Indeed, we found that *TYMS* knockdown led to the obvious upregulation of p21 and increase of SA-β-gal-positive cells in HCT116 cells (Fig. 4E and Fig. S4E).

To confirm the correlation between PLEK2 and TYMS, we analyzed their mRNA levels with the published next-generation sequencing data from CRC patient samples. PLEK2 expression was significantly correlated with the highly-exressed *TYMS* in human samples (Fig. S4F). Furthermore, ectopic expression of TYMS successfully reverted the upregulation of p21 and cell senescence in HCT116 cells with *PLEK2* silencing (Fig. 4F, G). TYMS overexpression also significantly restored the impaired HCT116 cell proliferation induced by the *PLEK2* knockdown (Fig. 4H). These data indicate that PLEK2 regulated cell proliferation and senescence of HCT116 via the expression of TYMS.

### PLEK2 interacted with YTHDF2 to regulate the *TYMS* mRNA stability

To determine how PLEK2 regulates TYMS expression, we first examined the effects of PLEK2 on the TYMS protein stability. *PLEK2* knockdown exhibited a comparable degradation of TYMS upon the cycloheximide (CHX) treatment in the CRC cells (Fig. S5A). The reduced TYMS proteins induced by PELI1 knockdown was also not reversed by the pre-treatment of MG132, a proteasome inhibitor (Fig. S5B).

**Fig. 5:**
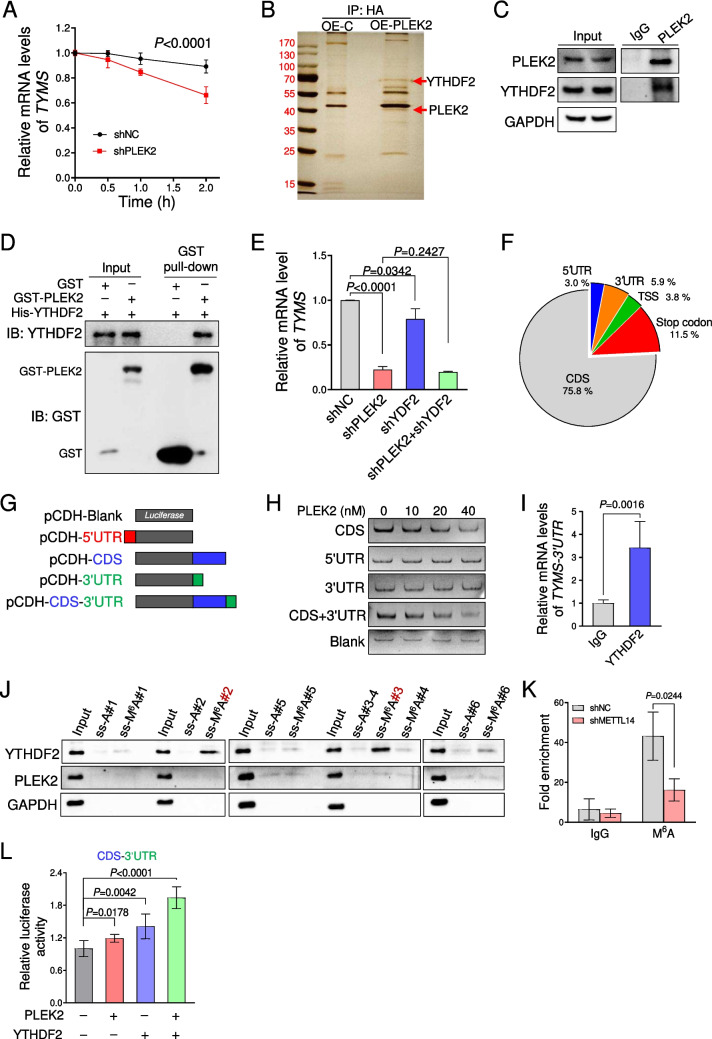
PLEK2-YTHDF2 complex promoted the *TYMS* transcript stability in a m^6^A‐dependent manner. (**A**) Quantitative PCR analysis of *TYMS* mRNA levels in HCT116 cells with *PLEK2* knockdown upon the treatment of actinomycin D (10 μg/ml). Data were presented as mean ± SD from three independent experiments. *P* value was determined by two-way ANOVA. shNC represents a non-targeting shRNA. (**B**) Proteomic study of PLEK2 interacting proteins in HCT116 cells transduced with retroviral constructs encoding HA-PLEK2 (OE-PLEK2) or empty vector (OE-C). Proteins immunoprecipitated using anti-HA were resolved by SDS-PAGE and visualized by silver staining followed by mass spectrometry analysis. (**C**) Co-IP analysis of endogenous YTHDF2 binding to PLEK2 in HCT116 cells. (**D**) GST pull-down assay of the physical interaction between PLEK2 (GST-Tagged) and YTHDF2 (His-Tagged) in vitro. (**E**) Quantitative PCR analysis of *TYMS* transcript in HCT116 cells transduced with retroviruses encoding indicated shRNAs. Data were presented as mean ± SD from three independent experiments. shNC represents a non-targeting shRNA. (**F**) Pie chart depicting the region distribution of PLEK2-binding sites identified by RIP-Seq. (**G**) Schematic representation depicting the Luciferase reporters herbing indicated regions of *TYMS* mRNA based on the pCDH plasmid. 5’UTR: 5’-untranslated regions, CDS: coding sequence, 3’UTR: 3’-untranslated regions. (**H**) EMSA assay showed direct binding of PLEK2 to the CDS region of *TYMS* transcripts as in G. (**I**) RIP analysis the 3’UTR of *TYMS* in HCT116 cells using YTHDF2 antibodies. Data were presented as mean ± SD from three independent experiments. (**J**) Immunoblotting of indicated proteins in HCT116 cells after RNA pull-down assay using single-stranded *TYMS* RNA with methylated (ss-A) or unmethylated adenosine (ss-M^6^A). #1-#6 indicated different sites of *TYMS* RNA as in Fig. S5J. (**K**) Quantitative PCR analysis of *TYMS* transcript in m.^6^A-MeRIP from HCT116 cells transduced with retroviruses encoding indicated shRNAs. Data were presented as mean ± SD from three independent experiments. shNC represents a non-targeting shRNA. (**L**) HCT116 cells carrying *Luciferase*-fusion CDS and 3’UTR regions of *TYMS* as in F were transduced with indicated lentivirus. *Luciferase* mRNA levels were analyzed after 48 h. OE-C represents overexpression of blank vector. Data were presented as mean ± SD from three independent experiments. See also Fig. S5

We then evaluate its mRNA expression upon the treatment of actinomycin D. *TYMS* showed a faster time-dependent decay in HCT116 cells transduced with PLEK2 shRNA than that of control groups (Fig. 5A). Considering that PLEK2 has been identified as a membrane and cytoskeletal protein[23], we speculated that other proteins interacts with PLEK2 to regulate *TYMS* mRNA stability. To this end, we performed immunoprecipitation of PLEK2 followed by mass spectrometry in HCT116 cells (Fig. 5B and Table S2). Among these identified proteins, YTHN6-Methyladenosine RNA Binding Protein 2 (YTHDF2) is an attractive candidate due to its role in the regulation of RNA stability in a m^6^A-dependent manner[34]. PLEK2 and YTHDF2 were reciprocally coimmunoprecipitated with each other in HEK293T cells with their overexpression (Fig. S5C). This was further confirmed by the co-IP assays of endogenous PLEK2 and YTHDF2 in HCT116 cells (Fig. 5C). Furthermore, GST pull-down assays showed that YTHDF2 were specifically retained in the presence of GST-PLEK2, confirming their physical interaction (Fig. 5D).

Indeed, YTHDF2 is also highly expressed in CRC (Fig. S5D). Silencing of *YTHDF2* led to the downregulation of *TYMS* via mRNA stability (Fig. 5E and Fig. S5E) and even the inhibitory proliferation of HCT116 cells (Fig. S5F), which phenocopied the effects of *PLEK2* knockdown on the HCT116 cells. However, *TYMS* did not show the further reduce in HCT116 cells transduced with PLEK2 shRNAs upon the *YTHDF2* knockdown (Fig. 5E). Similar findings were also observed in the proliferation assays of HCT116 cells (Fig. S5F-G). Moreover, we found that *TYMS* RNA stability were also impaired by the silencing of METTL14, one of critical components of RNA m^6^A modification as the “writer” (Fig. S5H). These data suggest that PLEK2 interacted with YTHDF2 to promote *TYMS* stability and proliferation of CRC cells in a m^6^A-dependent manner.

### PLEK2 and YTHDF2 cooperated to promote the *TYMS* mRNA stability in a m^6^A-dependent manner

Based on the above findings, we investigated whether PLEK2 function as an RNA-binding protein in CRC cells. We performed RNA immunoprecipitation sequencing using HA antibody in HCT116 cells with overexpression of HA-tagged PLEK2, and identified 1516 potential PLEK2-binding targets (Table S3). These RNA targets were substantially enriched in RNA transport/degradation and cell cycle pathways (Fig. S6A). Moreover, most of the PLEK2-binding sites were highly enriched on the CDS region of RNA targets including *TYMS* (Fig. 5F and Fig. S6B). To further confirm the direct binding of PLEK2 with *TYMS* mRNA, we constructed pCDH-Luciferase-based reporters bearing 5’-untranslated regions (5’UTR), coding sequence (CDS) and 3’UTR of *TYMS* mRNA respectively and incubated with PLEK2 protein *in vitro* (Fig. 5G). As expected, electrophoretic mobility shift assay results revealed that PLEK2 preferentially bound to the CDS region of *TYMS* mRNA (Fig. 5H).

We then analyzed the published RIP-seq data of YTHDF2 in HeLa cells [34], and found that 35.4% (537 out of 1,516) of the RIP targets of PLEK2 overlapped with that of YTHDF2 (Fig. S6C). Although *TYMS* is the common target of both proteins, YTHDF2 showed exclusive binding to the 3’UTR of *TYMS* mRNA that was demonstrated by RIP-qPCR analysis in HCT116 cells (Fig. 5I). Indeed, several potential m^6^A sites were observed in the *TYMS* mRNA containing the “DRACH” (D: A\G\U, R = A\G, H = A\U) consensus sequence [35] (Fig. S6D). To confirm that the binding of YTHDF2 to *TYMS* mRNA is m^6^A-dependent, we designed the methylated single-stranded RNA bait (ss-m^6^A) or unmethylated control RNA (ss-A) according to these potential m^6^A sites on the *TYMS* mRNA. RNA pull-down assay demonstrated that YTHDF2 specifically bound to two ss-m^6^A but not the corresponding unmethylated controls (ss-A) in the 3’UTR region of *TYMS* (Fig. 5J). Furthermore, we also performed m^6^A-MeRIP combined with *METTL14* knockdown. Quantitative PCR analysis of *TYMS* with primers targeting YTHDF2-binding sites showed that the enrichments of m^6^A sites were significantly reduced by *METTL14*-silencing in HCT116 cells (Fig. 5K). These data demonstrated that PLEK2/YTHDF2 binds to *TYMS* mRNA via an m^6^A-dependent manner.

To further confirm that the binding of PLEK2/YTHDF2 with *TYMS* mRNA mediates its stability, we constructed the pCDH-Luciferase-based reporters bearing CDS and 3’UTR of *TYMS* mRNA (pCDH-Luci-CDS-3'UTR, Fig. 5G). Single overexpression of PLEK2 or YTHDF2 mildly increased the luciferase activity in HCT116 cells transduced with pCDH-Luci-CDS-3'UTR, which was further enhanced by their combination (Fig. 5L). By contrast, similar approaches failed to detect any increase of luciferase activity in HCT116 cells transduced with pCDH-Luci-CDS or pCDH-Luci- 3'UTR (data not shown).

### Loss of PLEK2 inhibited AOM/DSS-induced colonic tumorigenesis *in vivo*

**Fig. 6:**
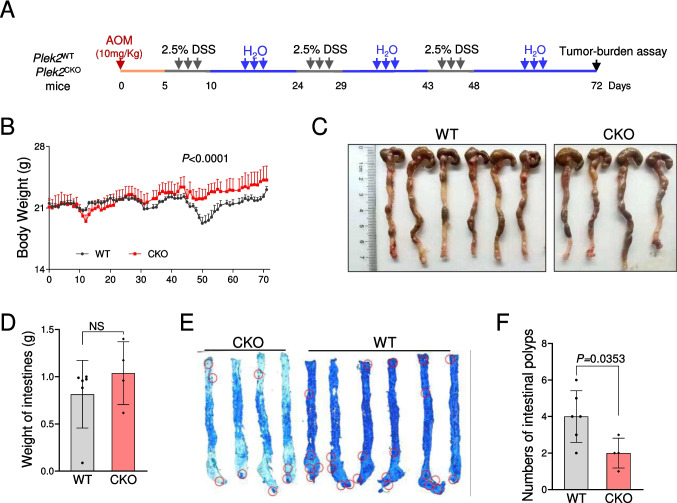
*Plek2* deletion reduced the intestinal tumorigenesis. (**A**) Schematic representation of inflammatory colorectal cancer mouse model with wild type (WT, *N* = 6) or *Plek2* conditional knockout (CKO, *N* = 4). (**B**) Statistical analysis of body weight from the mice as in A. (**C**) Representative image of colon in A. (**D**) Statistical analysis of intestines weight from the mice as in C. Each dot represents one mouse. Data were presented as mean ± SD. (**E**) Methylene blue staining of the colorectum from wild type (WT) and *Plek2*-CKO mice. (**F**) Statistical analysis of the numbers of intestinal polyps from the mice as in E. Each dot represents one mouse. Data were presented as mean ± SD. See also Fig. S6

To clarify the functional roles of PLEK2 in the tumorigenesis and development of CRC, we crossed *Plek2*^fl/fl^ mice with Vil1-cre transgenic mouse to generate an intestinal-specific knockout mouse model (Fig. S7A-D, referred to as CKO mice). *Plek2* deletion did not affect the intestinal function characterized by the comparable body and intestine weigh in CKO mice as WT mice (data not shown). We then utilized an AOM/DSS-induced colorectal tumor model, in which CKO or wild-type mice were given a single dose of the carcinogen azoxymethane (AOM) plus 3 cycles of 2.5% dextran sulfate sodium (DSS) (Fig. 6A). As expected, the body weight was reduced after DSS administration. However, CKO mice showed less susceptible to chronic inflammation, which was induced by AOM and DSS treatment, as compared to that of WT mice (Fig. 6B). Mice were sacrificed 72 days after AOM-DSS administration to analyze colonic tumor incidence and burden. Although there was no significant difference in colon length (Fig. 6C, D), the number of tumors was significantly reduced in CKO mice than that in WT mice (Fig. 6E, F). These results suggested that PLEK2 deficiency suppresses the colonic tumorigenesis in the colon.

## Discussion

TYMS is the main target of adjuvant chemotherapy drug (5-FU) in CRC, and its high expression is responsible for the long-term resistance to 5-FU treatment in CRC [4, 5, 36]. Our data demonstrated that PLEK2 and YTHDF2 cooperates to enhance *TYMS* mRNA stability in CRC, which is required for the CRC cell proliferation. *PLEK2* knockdown led to the downregulation of *TYMS* and consequently proliferative inhibition of CRC cells via cell senescence. In addition, PLEK2-mediated upregulation of *TYMS* in CRC cells via m^6^A modification, which evidenced by its decreased mRNA levels after *METTLE14* or *YTHDF2* silencing. It seems like that PLEK2 interacts with YTHDF2 to form a “protecting cap” through their binding to CDS and 3’-UTR respectively, which enhanced the *TYMS* mRNA stability.

YTHDF2 has been identified as a m^6^A “reader”, which binds and recognizes m^6^A methylation on mRNA to promotes its degradation with liquid–liquid phase separation. One recent study also revealed that YTHDF2 played a critical role in CRC cell proliferation via the degradation of m^6^A-modified GSK3β [21]. However, our data demonstrated that YTHDF2 is involved in the CRC proliferation via m^6^A-mediated *TYMS* stability. It is possible that *PLEK2* knockdown disrupted the “protecting cap”, leading to YTHDF2-mediated mRNA degradation of TYMS. This is consistent with the documented function of YTHDF2 that promotes mRNA degradation [16]. On the other hand, other YTH family members such as YTHDF1 and YTHDF3 may competitively bind and trigger *TYMS* mRNA degradation, since these YTH proteins share a set of common target mRNAs [14, 34].

p21 is a cyclin-dependent kinase suppressor factor, which is down-regulated in CRC [37, 38]. In agreement with this, upregulation of p21 inhibits the occurrence and invasion of CRC [39–41]. Our data also revealed that PLEK2 regulated p53-dependent p21 expression via TYMS. TYMS is an important enzyme involved in the de novo thymidylate synthesis, which is the rate-limiting step in DNA replication [42, 43]. *PLEK2* knockdown-mediated decrease of *TYMS* led to the suppressed DNA replication in CRC cells. This is associated with the activation of p53-p21 signaling and eventually cell senescence in CRC cells.

It is worth noting that PLEK2 was highly expressed in colorectal CCSCs. Consistently, PLEK2 had been proven to play roles in the proliferation of pancreatic cancer stem cells [44]. CCSCs represent a sub-type of tumor cells attributed to critical steps in cancer including tumor propagation, therapy resistance, recurrence and in some cases metastasis [45]. *PLEK2* silencing impaired the proliferation and stemness of CCSCs, which may account for the suppressed metastasis and invasion of CRC cells with *PLEK2* knockdown *in vitro* and *in vivo*. Given that PLEK2 is required for CCSCs, targeting PLEK2 inhibition has the potential to eliminate CCSCs via the reduction of self-renewal accompanied by its differentiation into cancer cells.

Taken together, our study identified PLEK2 as a key regulator for the progress of CRC, and demonstrated that PLEK2-YTHDF2 cooperates to protect *TYMS* mRNA from degradation.

## Materials and methods

### Mice

The following mice were maintained on the C57BL/6 genetic background. To specifically delete *Plek2* in colonic tissue, *Plek2*^flox/flox^; Vil1-cre mice (*Plek2*^CKO^) were bred using *Plek2*^flox/flox^ and *Vil1*-cre mice purchased from Jackson laboratory. C57BL/6 recipient mice were purchased from SPF Biotechnology Co., Ltd. (Beijing, China). Experiments were performed on age- and sex-matched cohorts. All animal studies were performed in accordance with the Guidelines for the Care and Use of Laboratory Animals and were approved by the Institutional Animal Care and Use Committees at Shandong University (#20021).

### Cell culture

The HCT116, HT29 CRC cell lines and HEK293T cells were purchased from the Cell Bank of the Shanghai Institute for Biological Sciences, Chinese Academy of Science. These cell lines were cultured in DMEM medium (BasalMedia, L110KJ) supplemented with 10% FBS (SeraPure, SE141-500). CRC stem cell cells (CCSCs) derived from HCT116 cells were kindly provided by Huili Hu Lab, ShanDong university, and were cultured in DMEM-F12 medium (BasalMedia, L320KJ) supplemented with B27 (Thermo, 17504044), N2 (Thermo, 17502048), EGF (Proteintech, HZ-1326) and bFGF (Proteintech, HZ-1285).

### Plasmids construction and cell transduction

Cloning of shRNA into the pLKO.1 lentivirus vector was performed as previously described [24], and shRNA oligonucleotides were shown in Table S4. Plasmids encoding FLAG tagged YTHDF2, HA tagged PLEK2 and FALG tagged TYMS-NLS were cloned into pCDH lentivirus vector, the 5’UTR, CDS and 3’UTR of TYMS were heterologous expressed with luciferase in pCDH-Luciferase vector. Viral packaging and injection were conducted as described previously [46]. For GST pull-down assay, the full-length sequence of PLEK2 was cloned into pGEX-6P-1 containing a GST tag and the primers were shown in Table S4.

### Co-immunoprecipitation and mass spectrometry analysis

Cell lysates were obtained and immunoprecipitated with beads conjugated with indicated antibodies as previously described methods [47]. The beads-protein conjugates were then washed with IP buffer five times and boiled at 95 °C in 4 × NuPAGE™ LDS Sample Buffer (Thermo, NP0008) for 10 min. Aliquots were analyzed by immunoblotting, in parallel, proteins were identified using mass spectrometry (BGI). The antibodies used in immunoprecipitation were listed in Table S5. The interacting proteins were determined as listed in Table S2.

### Azoxymethane/dextran sulfate sodium (AOM/DSS) model

*Plek2*^CKO^ and *Plek2*^WT^ mice were given a single dose of the carcinogen azoxymethane (AOM, 10 mg/kg, GLPBIO, GC19445,) plus 3 cycles of 2.5% dextran sulfate sodium (DSS, GLPBIO, GC19829) to induce colon cancer, the weight of mice was monitored during drug administration. Methylene blue dye (GLPBIO, GC47663) was used for staining of intestinal tumors. Briefly, mouse small intestines were collected and flushed with cold PBS to remove the faecal contents. Intestines were then opened and rinsed in 1% methylene blue dye for 1 h at room temperature. The excessive methylene blue dye was washed out by rinsing the intestines with PBS overnight. The tumors number was measured and assessed via measuring the deep stains in intestines and recorded with photographs.

### Colony formation assay

For the colony formation assay, 50 cells were seeded in six-well plates. After 10–15 days, the cells were stained with crystal violet (Beyotime, C0121). The number of colonies was countered for five representatives’ fields and the experiments were repeated for three times.

### Senescence-associated β-galactosidase staining

HCT116 or HT29 cells to be detected were cultured in DMEM medium containing doxorubicin (Beyotime, SC0159, 0.3 μM) and cultured for 24 h. Then cells were washed with PBS for 3 times and stained with freshly prepared SA-β-Gal staining solution following the protocol provided by the manufacturer (Beyotime, C0602). The stained cells were photographed and the percentage of senescence cells was quantified by calculating the percentage of SA-β-Gal-positive cells in randomly selected fields (*n* = 3).

### RNA immunoprecipitation (RIP) assays

RIP was conducted with the RIP Immunoprecipitation Kit (Geneseed, P0101) according to the manufacturer’s instructions. Briefly, 1 × 10^7^ HCT116 cells were collected and lysed by lysis buffer. Agarose beads coated with 2 µg of specific antibodies against human YTHDF2 or PLEK2 were incubated with prepared cell lysates overnight at 4 °C. RNA was eluted by 15 µL of RNase-free water in the following steps, the relative interaction between YTHDF2 or PLEK2 and TYMS transcripts was determined by qPCR and normalized to the input.

### CCSCs spheroid formation assays

The spheroid-derived CCSCs from HCT116 cells were cultured in Costar ultralow attachment flasks (Corning) in DMEM/F12 medium containing N2 supplement (1/100), B27 supplement (1/50), EGF (40 ng/ml), and bFGF (20 ng/ml). Spheres were dissociated using 0.25% trypsin–EDTA, then the single cells transduced with the *PLEK2* shRNA or overexpression retrovirus and cultured for 5–7 days. Spheres with a diameter over 50 μm were counted. The single cells were cultured in DMEM containing 3% FBS for 48 h to induce cell differentiation.

### Electrophoretic mobility shift assay (EMSA) for protein-DNA binding

The PLEK2 protein was purified *in vitro* using bacterially expressed proteins. The TYMS gene CDS and 3’UTR was PCR amplified form pCDH plasmid expressing luciferase protein with primers listed in Table S3. And the 5’UTR of TS gene and the empty carrier vector were linearized by the corresponding enzyme. Binding reactions were conducted in a 20 μl binding buffer (Beyotime, GS005) containing 100 ng of indicated DNA and different concentrations of PLEK2 protein at 37℃ for 15 min. The samples were then separated in a 4% EMSA page gel (WSHT, E301E4T) in the ice at 100 V for 1 h. After that, gels were stained with EB (5 g/ml) for 20 min, visualized by UV illumination with 312 nm, and photographed using Gel Imaging.

### RNA-pull down

The desthiobiotin-labeled single-stranded RNA containing methylated or unmethylated adenosine were synthesized by Sangon Biotechnology (shanghai) Co., Ltd and listed in Table S6 in the supporting Information. RNA pulldown assays were performed as the PureBinding ® RNA–Protein pull-down Kit (Geneseed, P0201) described. Briefly, up to 100 pM of RNA incubated with 50 µL of Streptavidin Magnetic Beads and 2 mg of HCT116 cells protein lysates. Finally, the eluted RNA-binding protein complexes were boiled and assay with anti-YTHDF2 antibody.

### RIP-seq

1X10^7^ HCT116 cells transduced with retroviruses encoding HA tagged PLEK2 and empty vector were collected and lysed according to the instructions of RIP Immunoprecipitation Kit (Geneseed, P0101). Briefly, Agarose beads coated with 2 µg of HA antibodies against human PLEK2 were incubated with prepared cell lysates overnight at 4 °C. RNA was eluted by 15 µL of RNase-free water in the following steps, then ribominus rRNA were depleted by the rRNA depletion kits (Thermo, K155001), for high-throughput sequencing, the libraries were prepared following the manufacturer’s instructions and applied to Illumina NovaSeq 6 000 system for 150 nt paired-end sequencing (Novogene).

### MeRIP -qPCR

1X10^7^ HCT116 cells transduced with retroviruses encoding PLEK2 shRNA or empty vector were collected and the total RNA was extracted by Trizol. then, the Methylated RNA Immunoprecipitation (MeRIP) was performed according to the instructions of the MeRIP Kit (Bersinbio, Bes5203). Briefly, 100 μg total RNA was fragmented into ~ 300-nucleotide (nt) fragments using ultrasound, the fragmented RNA was then incubated with 5 μg of anti-m6A antibody or control IgG in a vertical mixer at 4 °C for 4 h. Next, the Protein A/G magnetic beads sealed with BSA were added to the mixture and incubated for 1 h with continuous shaking at 4 °C. The m6A-modified RNA was competitively eluted using 150 μL of elution buffer and Phenol–Chloroform-isopentanol mixture (25:24:1). The upper water phase was collected and added 1 μl of glycogen, 20 μl of sodium acetate and 400 μl of 100% ethanol at − 80 °C overnight. The m6A-bound RNA was calculated by qPCR and the corresponding m6A enrichment was calculated by normalizing to the input.

### Statistical analysis

Statistical analyses were performed with unpaired two-tailed Student’s t-test except were indicated otherwise using Prism (GraphPad). The *P* values < 0.05 were considered significant.

## Supplementary Information

Below is the link to the electronic supplementary material.

Supplementary file1 (XLSX 472 KB)

Supplementary file2 (XLSX 133 KB)

Supplementary file3 (XLSX 90 KB)

Supplementary file4 (DOCX 2555 KB)

## Data Availability

The RIP-seq data that support the findings of this study are openly available in [Gene Expression Omnibus. GEO] at [https://www.ncbi.nlm.nih.gov/geo/query/acc.cgi?acc=GSE252234], reference number [GSE252234] and the RNA-seq data that support the findings of this study are openly available in GEO [https://www.ncbi.nlm.nih.gov/geo/query/acc.cgi?acc=GSE252372], reference number [GSE252372]. The other data generated in this study are available in the article and its supplementary data files or upon request from the corresponding author.
