# Supplementary material for: Pleckstrin-2 promotes the progression of colorectal cancer via YTHDF2-mediated TYMS mRNA stability
Source: Cell Mol Life Sci. 2025 Jul 19;82(1):284. doi: 10.1007/s00018-025-05782-x (PMC12276184; doi:10.1007/s00018-025-05782-x)
Supplement: Supplementary file 4 — Supplementary file4 (DOCX 2555 KB) [file 18_2025_5782_MOESM4_ESM.docx]

**Supplemental Information**

**Supplemental Materials and Methods**

**Generation of *Plek2* conditional knockout mice**

We made *Plek2* conditional knockout mice via CRISPR/Cas9-mediated genome engineering. Briefly, Cas9 mRNA, sgRNA and donor were co-injected into zygotes. sgRNAs (5’-CCAATTAGAGGATTCAAAAGTGG-3’, 5’- ACTGTTACACCCGTCATAGATGG-3’) directed Cas9 endonuclease cleavage in before and after exon 3 and created a double-strand break. LoxP sites were inserted into before and after exon 3 respectively by homologous recombination. LoxP sites were verified by DNA sequencing.

Chimeric mice were generated by standard methods. *Plek2* deletion was achieved by crossing with transgenic Vil1-cre mice purchased from JAX Lab (B6. Cg-Tg (Vil1-cre)1000Gum/J), by which Exon 3 floxed by loxP sites were deleted in intestinal epithelial cells. This led to the disruption of Plek2 expression. *Plek2*^fl/fl^ littermate mice were genotyped by PCR with primers *Plek2*-F (5’- GGTGACCACAATTAGACTTGACTTG-3’) and *Plek2*-R (5’- GACAGTCTTTGTACTATGGGCCAC-3’).

Excision after Vil1-Cre recombination was confirmed by PCR with primers to detect a portion that remains post excision (*Plek2*-F (5’- GGTGACCACAATTAGACTTGACTTG-3’) and *Plek2*-R (5’- GACAGTCTTTGTACTATGGGCCAC-3’; *Vil1*-Cre-F (5’- CAGCATTGCTGT CACTTGGTC-3’) and *Vil1*-Cre-R (5’-ATTTGCCTGCATTACCGGTCG-3’).

**Cell migration**

The wound healing assay was used to analyze the cell migration. Cell supernatants knocking down of *PLEK2* were discarded and PBS washed cells three times. Then cells were scratched by a peptide and incubated in DMEM medium containing 1% FBS. The wound healing area was observed and calculated at 24 h or 48 h after scratching.

**Mouse xenograft model of CRC tumors**

To analyze the effect of PLEK2 on tumor growth in vivo. 2×10^6^ HCT116 cells (2×10^6^/100 μl) transduced with retroviruses encoding *PLEK2* shRNA or non-targeting shRNA, were mixed with 100 μl of Matrigel basement membrane matrix (Corning, 354248) and injected subcutaneously into the right armpit of nude mice. The tumor size was measured every other day from Day 7, and the volume was calculated as Volume =(Length×Width^2^) ×0.52[1]. The mice were euthanized 28 days after implantation, and the tumors were removed for gross examination.

**Immunoblotting**

Cells were lysed in RIPA buffer (Beyotime, P0013B) with protease and phosphatase inhibitor mixture (Beyotime, P1045) for 30 min, then centrifuged at 12000 rpm, 4 °C. The protein expression levels were determined by staining with primary antibodies. The antibodies used in this study were listed in Table S5.

**RNA stability**

Actinomycin D (MCE, HY-17559, 10 μg/ml) was added to the PLEK2, YTHDF2 or METTL14 stable knockdown HCT116 cells, then cells were collected after incubation at the indicated times (0, 0.5, 1, 2 h) and qRT-PCR was performed.

**mRNA extraction and Quantitative RT-PCR**

Total RNA from CRC cells was extracted using the Trizol reagent (Invitrogen, 15596026CN) according to the manufacturer’s instructions. RNA quality and quantity were determined using a Nano Drop and Agilent 2100 bioanalyzer (Thermo). RNA was reversely transcribed into cDNA using RT reagent kit (TaKaRa, RR047). A SYBR Green PCR kit (TaKaRa, RR420) was used for quantitative real-time PCR and results were quantified with an Applied Biosystem System (ABI) with appropriate primers. The human housekeeping gene GAPDH was used as the RNA-loading control. Gene expression was determined by the delta CT method (2^-∆∆Ct^). ∆Ct = (Ct target gene – Ct housekeeping). The primers and reagents used in this study were listed in Table S4.

**Cell cycle analysis**

Cells were harvested and washed with cold PBS and fixed in 70% ethanol overnight at -20°C. Cells were re-suspended in PBS containing 100 μg/ml RNase A and 5 μg/ml PI at 37°C for 0.5 h. Cell cycle distribution was measured by flow.

**Cell proliferation assay**

Cell viability was analyzed using CCK8 assays (Dojindo, CK04) as described previously[2], Briefly, 2x10^3^ HCT116 or HT29 cells per well were seeded in 96-well plates for a given time. At the indicated time points, 100 µl fresh medium containing 1/10 volume of CCK8 was added to each well. Absorbance at 450 nm was measured after incubation at 37°C for 2 h. Cell proliferation was also analyzed using EdU Cell Proliferation Kit (Beyotime, C0078S) according to the manufacturer’s instructions.

**Immunohistochemistry**

The tissue microarray from CRC patients was dewaxed and heated in antigenic repair solution for 15 min. Then incubated with PLEK2 antibody as described[3]. The tissue microarray was scanned by a panoramic slide scanner and quantitatively analyzed by Quant Center software. H-Score values were calculated by the following: H-Score = (percentage of weak intensityx1) + (percentage of moderate intensityx2) + (percentage of strong intensityx3).

**GST pull-down assay**

pGEX-6P-1-GST and pGEX-6P-1-GST-PLEK2 plasmids were transfected into E. coli respectively. Proteins were expressed and purified following the instructions of GST-tag Protein Purification Kit (Beyotime, #P2262). His-YTHDF2 protein were purchased from MCE (#HY-P702576). 25 µg of GST and GST-PLEK2 fusion protein were incubated with 15 µg of His-YTHDF2 respectively, in 50 µL of glutathione agarose overnight at 4°C. The bound proteins were analyzed by immunoblotting.

**Supplemental Tables**

**Table S1. RNA-seq analysis of HCT116 cells transduced with retroviruses encoding *PLEK2* shRNA and non-targeting control.**

**Table S2. Identified proteins from co-immunoprecipitation with PLEK2 antibody in HCT116 cells.**

**Table S3. Transcripts identified by RIP-Seq with PLEK2 antibody in HCT116 cells and the overlapped genes with YTHDF2 RNA targets from published data.**

**Table S4. Primer sequences used in this study.**

| **Primer** | **species** | **Sequence** |
| --- | --- | --- |
| PLEK2-F | Homo | CCCTGGTGGACTGGCTATC |
| PLEK2-R | Homo | CGAATGGCTCCCATGCTTCG |
| GAPDH-F | Homo | CTGGGCTACACTGAGCACC |
| GAPDH-R | Homo | AAGTGGTCGTTGAGGGCAATG |
| METTL14-F | Homo | AGTGCCGACAGCATTGGTG |
| METTL14-R | Homo | GGAGCAGAGGTATCATAGGAAGC |
| TYMS-F | Homo | CGCTACAGCCTGAGAGATGA |
| TYMS-R | Homo | ACTCCCTTGGAAGACAGCTC |
| p21-F | Homo | CGACTGTGATGCGCTAATGG |
| p21-R | Homo | GTGGTGTCTCGGTGACAAAG |
| Ki67-F#1 | Homo | GCCTGCTCGACCCTACAGA |
| Ki67-R#1 | Homo | GCTTGTCAACTGCGGTTGC |
| Ki67-F#2 | Homo | AGAAGAAGTGGTGCTTCGGAA |
| Ki67-R#2 | Homo | AGTTTGCGTGGCCTGTACTAA |
| TYMS-RIP-F#1 | Homo | AAATGTAACTGTGCCAGTTC |
| TYMS-RIP-R#1 | Homo | CATTCTCCTCACTTTGTTCA |
| TYMS-RIP-F#2 | Homo | CAAGCTATTCCCTCAAATCT |
| TYMS-RIP-R#2 | Homo | AACTGAGCAGATAAGTGGCA |
| METTL14-shRNA#1 | Homo | CCATGTACTTACAAGCCGATA |
| METTL14-shRNA#2 | Homo | GCCGTGGACGAGAAAGAAATA |
| PLEK2-shRNA | Homo | GCTGGTGTACTACAAGCTTGA |
| TYMS-shRNA#1 | Homo | CCCTGACGACAGAAGAATCAT |
| TYMS-shRNA#2 | Homo | CAGGTGACTTTATACACACTT |
| YTHDF2-shRNA#1 | Homo | TCTGGATATAGTAGCAATTAT |
| YTHDF2-shRNA#2 | Homo | GCTACTCTGAGGACGATATTC |
| pCDH-TYMS-CDS-F | Homo | ATGCCTGTGGCCGGCTCGGAGCTGC |
| pCDH-TYMS-CDS-R | Homo | CTAAACAGCCATTTCCATTTTAATA |
| pCDH-TYMS-3’UTR-F | Homo | GGTGCTTTCAAAGGAGCTCG |
| pCDH-TYMS-3’UTR-R | Homo | TTTACTTAAGGAGAGTTCTT |
| pGEX-6P-1-PLEK2-F | Homo | ATGGAGGACGGCGTGCTCAAGG |
| pGEX-6P-1-PLEK2-R | Homo | TCATGTTAGCTTTTTGATAGCT |
| TYMS-F(M^6^A-IP-qPCR) | Homo | AAAAATCTGTCCGTGACCTA |
| TYMS-F(M^6^A-IP-qPCR) | Homo | ATAACCTCAGCATTGTCAGA |

**Table S5. Antibodies and commercial reagents used in this study.**

| **Antibody** | **Brand** | **Cat#** |
| --- | --- | --- |
| PLEK2 | Proteintech | 11685-1-AP |
| GAPDH | Abcam | ab128915 |
| TYMS | Proteintech | 15047-1-AP |
| Normal Rabbit IgG | CST | 2729S |
| p21 PolyAb | Proteintech | 10355-1-Ap |
| Phospho-Rb(Ser807/811) | CST | 8516 |
| Rb | CST | 9309 |
| E2F1 | Abcam | ab179445 |
| Phospho-p53(S15)  p53 | CST  CST | 9284S  2527 |
| Anti-HA-tag mAb | MBL | M180-3 |
| LaminB1 | Proteintech | 66095-1-Ig |
| YTHDF2 | Proteintech | 24744-1-AP |
| Alpha-Tubulin | Affinity | AF7010 |
| Histone H3 | CST | 4499 |
| Anti-HA-Tag | Affinity | T0050 |
| Anti-DDDK-tag-FLAG | MBL | M185-3 |
| Goat Anti-Rabbit IgG H&L (Alexa Fluor® 568) | Abcam | ab175471 |
| HRP-Goat Anti-Rabbit IgG | Jackson immuno | 111-035-144 |
| HRP-AffiniPure Goat Anti-Mouse IgG | Jackson immuno | 115-035-003 |
| **Reagents** |  |  |
| β-galactosidase Assay Kit | Beyotime | C1115 |
| PureBinding ® RNA-Protein pull-down Kit | Geneseed | P0201 |
| RNA Immunoprecipitation Kit | Geneseed | P0101 |
| B27 | Thermo | A1486701 |
| N2 | Thermo | 17502048 |
| EGF | Proteintech | HZ-1326 |
| bFGF | Proteintech | HZ-1285 |
| BeyoClick™ EdU Kit | Beyotime | C0078S |
| GST-tag Protein Purification Kit | Beyotime | P2262 |
| His-YTHDF2 | MCE | HY-P702576 |

**Table S6. Biotin-labeled TYMS RNA probes sequences used in this study.**

| **Primer** | **species** | **Sequence** |
| --- | --- | --- |
| ss-A#1 | Homo | AAGGAGCUCGAAGGAUAUUGUCAGUCUUUAGGGGUUGGGCUGGAU |
| ss-M6A#1 | Homo | AAGGAGCUCGAAGGAUAUUGUC/6-MerA/GUCUUUAGGGGUUGGGCUGGAU |
| ss-A#2 | Homo | AAAAAUCUGUCCGUGACCUAUCAGUUAUUAAUUUUUAAGGAUGUU |
| ss-M6A#2 | Homo | AAAAAUCUGUCCGUGACCUAUC/6-MerA/GUUAUUAAUUUUUAAGGAUGUU |
| ss-A#3 | Homo | AUAACCUCAGCAUUGUCAGAUACCCUCAGUGAGUUAACUCAAAGC |
| ss-M6A#3 | Homo | AUAACCUCAGCAUUGUCAGAUACCCUC/6-MerA/GUGAGUUAACUCAAAGC |
| ss-M6A#4 | Homo | AUAACCUCAGCAUUGUC/6-MerA/GAUACCCUCAGUGAGUUAACUCAAAGC |
| ss-A#5 | Homo | GAUGGUGUUACUCAGCUCCCUCAGAUUUGAGGGAAUAGCUUGUGA |
| ss-M6A#5 | Homo | GAUGGUGUUACUCAGCUCCCUC/6-MerA/GAUUUGAGGGAAUAGCUUGUGA |
| ss-A#6 | Homo | CUGUACUGCCACUUAUCUGCUCAGUUCCUUCCUAAAAUAGAUUAA |
| ss-M6A#6 | Homo | CUGUACUGCCACUUAUCUGCUC/6-MerA/GUUCCUUCCUAAAAUAGAUUAA |

**Supplemental Figures**


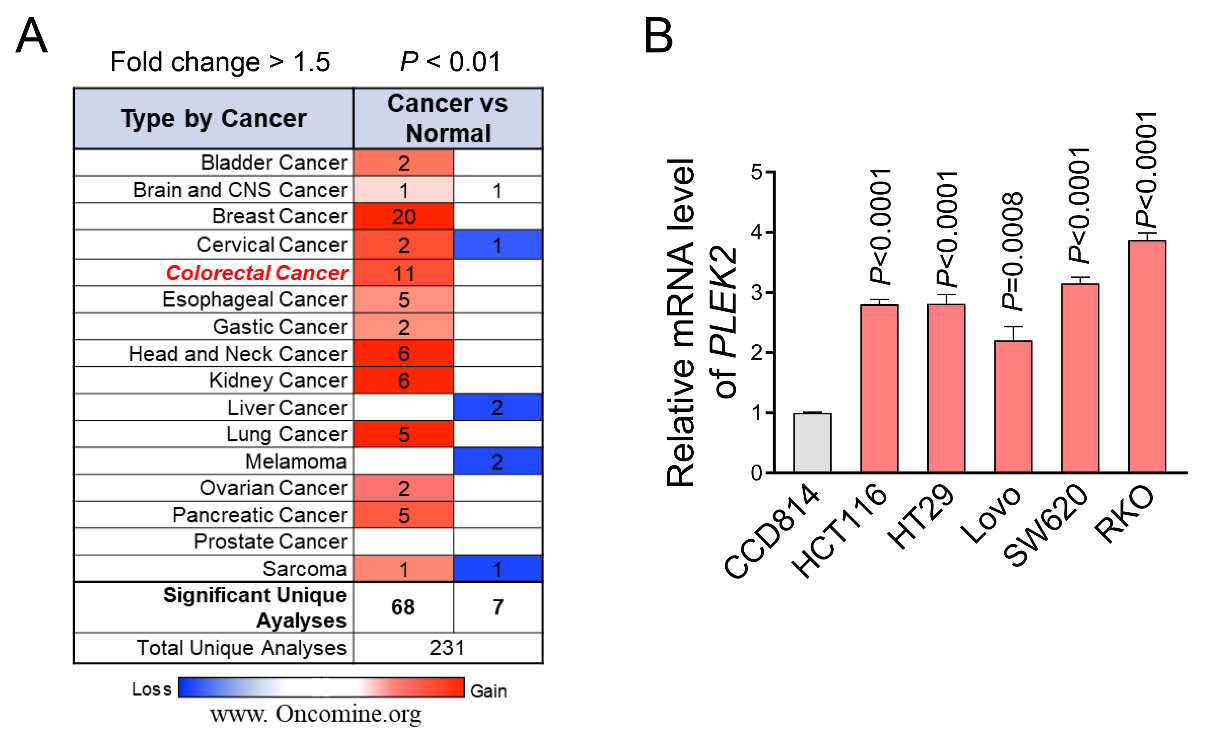


**Figure S1. PLEK2 is highly expressed in CRC. Related to Fig. 1**

(**A**) PLEK2 pan-cancer analysis using the Oncomine database (http://www.oncomine.org). (**B**) Quantitative PCR analysis of *PLEK2* mRNA levels in Human normal colonic epithelial cells (CCD841) and indicated CRC cell lines. Data were presented as mean±SD from three independent experiment.


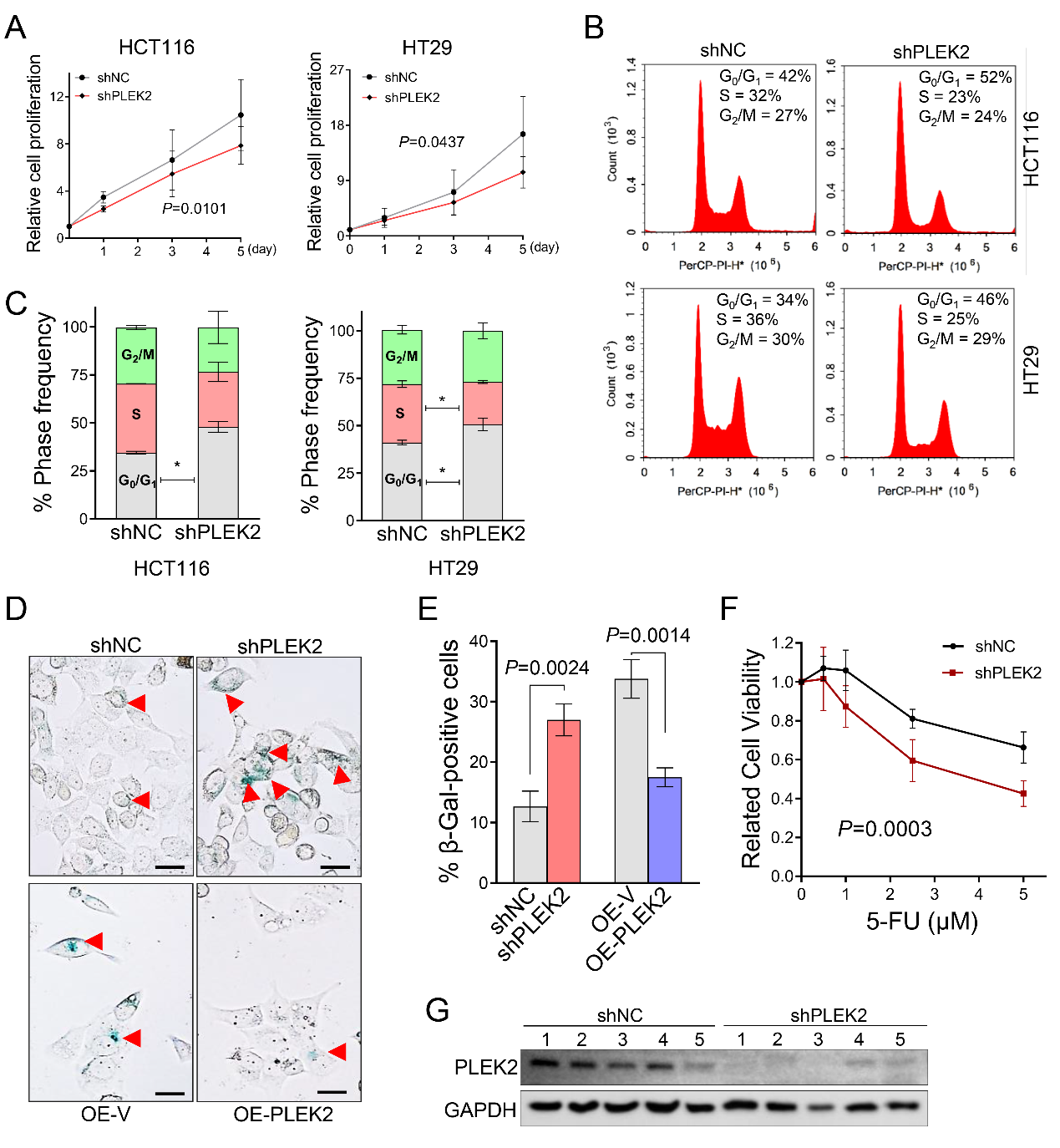


**Figure S2. Silencing of PLEK2 promoted cellular senescence in CRC cells. Related to Fig. 2**

(**A**) Quantification of cell proliferation in HCT116 and HT29 cells transduced with retroviruses encoding indicated shRNAs. shNC represent a non-targeting shRNA. Data were presented as mean±SD from three independent experiment. *P* value was determined by two-way ANOVA. (**B-C**) Cell cycle distribution in HCT116 and HT29 cell transduced with retroviruses encoding indicated shRNAs for 30 h. shNC represent a non-targeting shRNA. Data were presented as mean±SD from three independent experiment. (**D**) Representative images of SA-β-gal staining in HT29 cells transduced with indicated retroviruses treated in the presence of Doxorubicin (0.3 μM) for 24 h. Red arrows indicated senescent cells. OE-C represents overexpression of blank vector, and shNC represents a non-targeting shRNA. Scale bars, 25 µm (**E**) Quantification of SA-β-gal positive cells in D. Data were presented as mean±SD from three independent experiments. (**F**) Statistical analysis of cell proliferation in HCT116 cells transduced with indicated shRNAs upon the treatment of 5-Fluorouracil (5-FU). Data were presented as mean±SD from three independent experiments. *P* value was determined by two-way ANOVA. (**G**) Immunoblotting analysis of PLEK2 levels in the tumor cells of node models. GAPDH was used as the loading control.


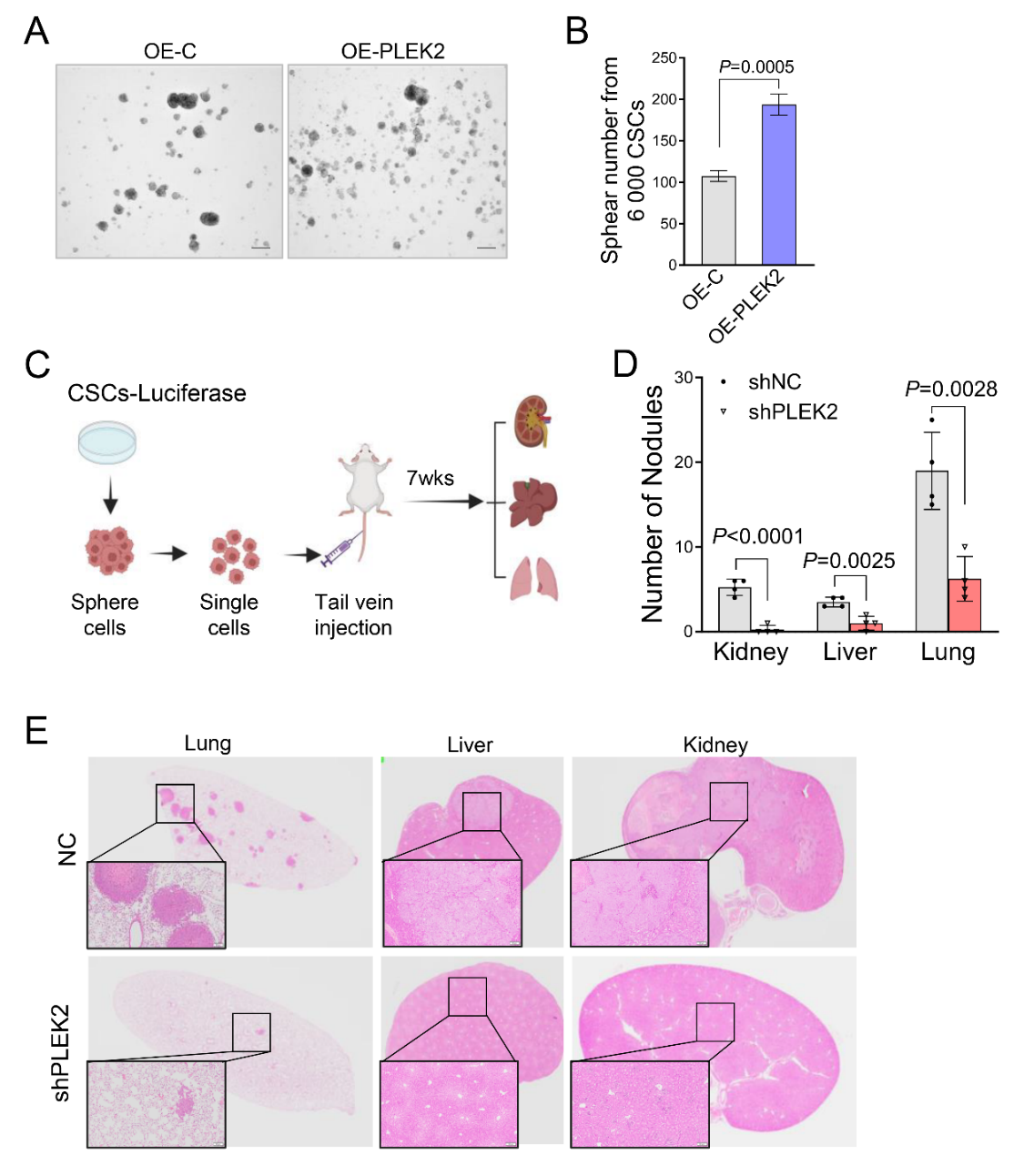


**Figure S3. PLEK2 overexpression promoted the tumor metastasis of HCT116 CCSCs. Related to Fig. 3**

(**A**) Sphere formation activity of HCT116-CCSCs transduced with indicated retroviruses and cultured for 7 days. OE-C represents overexpression of blank vector. Scale bars, 200 µm. (**B**) Quantitation Sphere numbers derived from 6000 CCSCs as in A. Data were presented as mean±SD from three independent experiments. (**C**) Schematic representation of CCSCs metastasis assay. This image was created with BioRender.com. (**D**) Statistical analysis of the tumor nodules in kidney, liver and lung from indicated mice as in Fig. 3H. Each dot represents one mouse. Data were presented as mean±SD. shNC represents a non-targeting shRNA**.** (**E**) Representative HE staining of kidney, liver and lung from the mice as in Fig. 3H. shNC represents a non-targeting shRNA**.** Scale bars, 100µm.

**
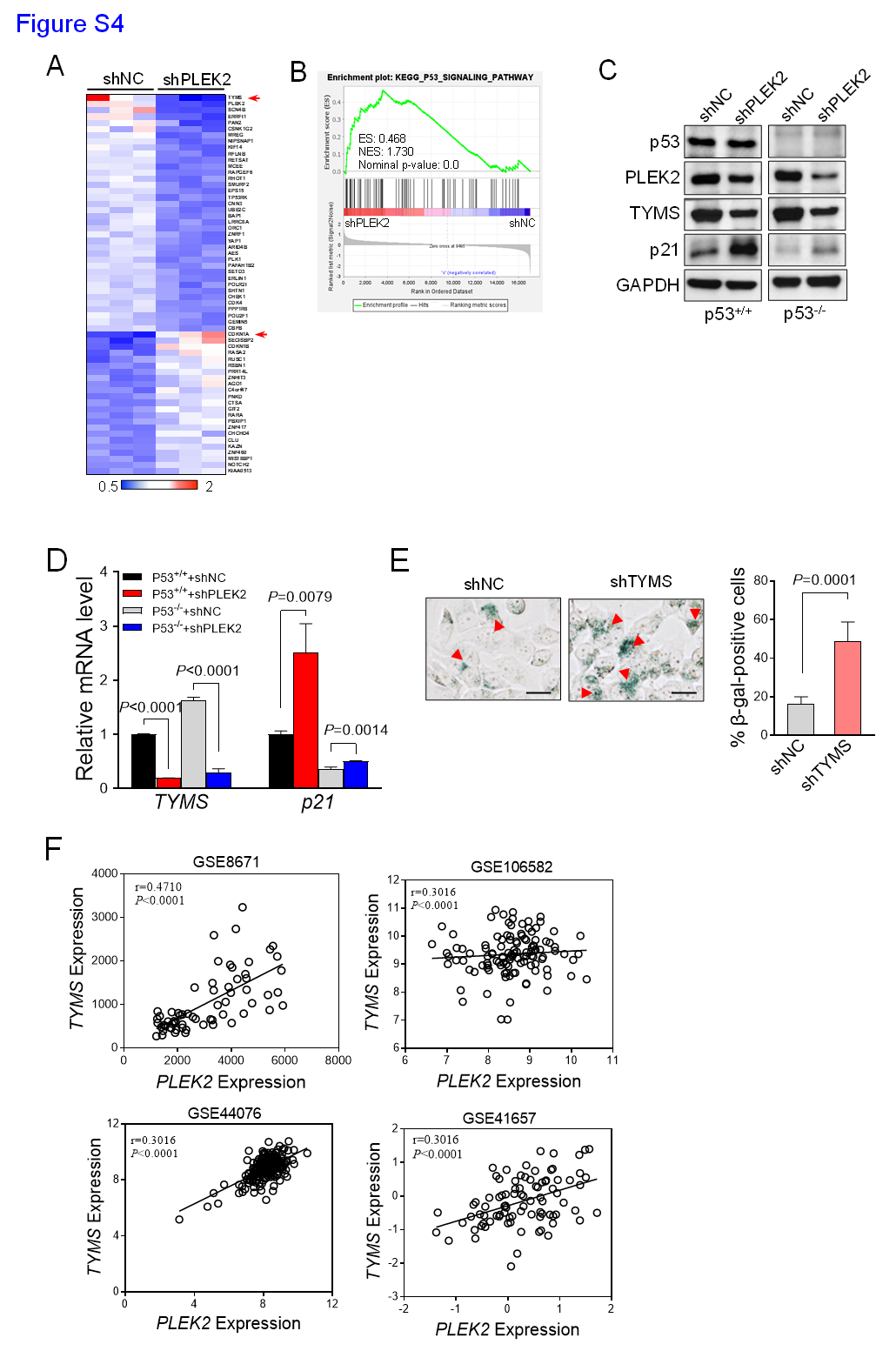
**

**Figure S4. Down-regulation of p21 by PLEK2 shRNA is p53-dependent. Related to Fig. 4**

(**A**) Heat map showing differentially expressed proteins in HCT116 cells transduced with retroviruses encoding indicated shRNAs. shNC represents a non-targeting shRNA. (**B**) Gene Set Enrichment Analysis of p53 signaling pathway in HCT116 cells transduced with retroviruses encoding *PLEK2* shRNAs. shNC represents a non-targeting shRNA. (**C**) Immunoblotting analysis of indicated proteins in p53^+/+^ and p53^-/-^ HCT116 cells transduced with retroviruses encoding *PLEK2* shRNAs. shNC represents a non-targeting shRNA. GAPDH was used as the loading control. (**D**) Quantitative PCR analysis of indicated mRNAs in cells as in D. (**E**) Representative images (left) and quantitative analysis (right) of SA-β-gal staining in HCT116 cells with retroviruses encoding *TYMS* shRNAs. shNC represents a non-targeting shRNA. Data were presented as mean±SD from three independent experiments. Scale bars, 25 µm. (**F**) RNA-seq expression profiles of *PLEK2* and *TYMS* in colorectal cancer tissues from public databases (GSE8671, GSE106582, GSE44076 and GSE41657).

**
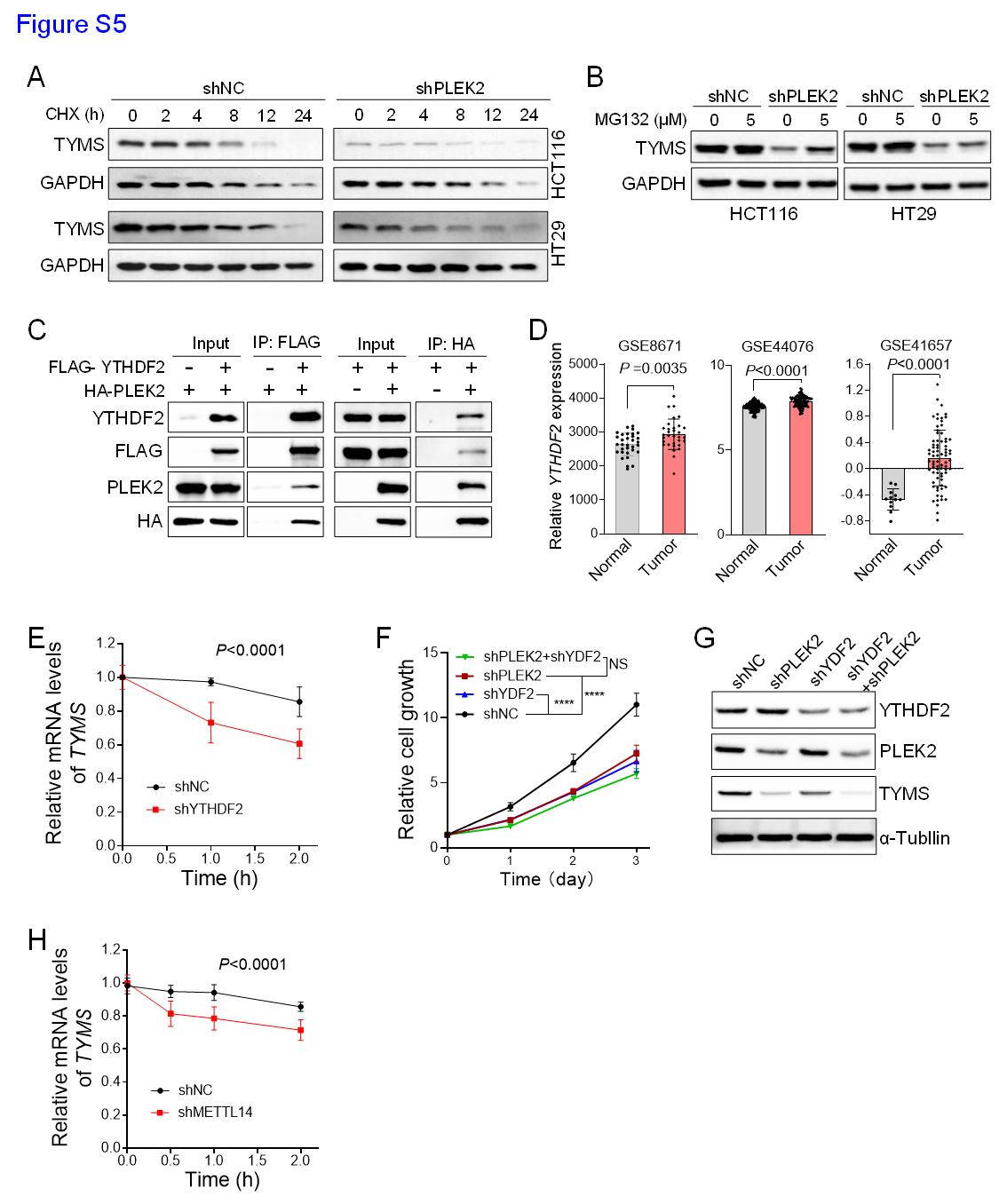
**

**Figure S5. PLEK2 interacted with YTHDF2 in CRC. Related to Fig. 5**

(**A**) Immunoblotting analysis of TYMS in HCT116 and HT29 cells with indicated shRNAs upon cycloheximide (CHX, 10 μM) treatment. shNC represents a non-targeting shRNA. (**B**) Immunoblotting analysis of TYMS in HCT116 and HT29 cells with indicated shRNAs upon MG132 treatment for 2 h. shNC represents a non-targeting shRNA. (**C**) Co-IP analysis of PLEK2 binding to YTHDF2 in HEK293T cells co-transduced with YTHDF2 (FLAG-tagged) and PLEK2 (HA-tagged). (**D**) RNA-seq expression profiles of *YTHDF2* in colorectal cancer and paired para cancerous tissues from public databases (GSE8671, GSE44076 and GSE41657). (**E**) Quantitative PCR analysis of *TYMS* mRNA levels in HCT116 cells with *YTHDF2* knockdown upon the treatment of actinomycin D (10 μg/ml). Data were presented as mean±SD from three independent experiments. *P* value was determined by two-way ANOVA. shNC represents a non-targeting shRNA. (**F**) Statistical analysis of cell proliferation in HCT116 cells transduced retroviruses encoding indicated shRNAs. shNC represent a non-targeting shRNA. Data were presented as mean±SD from three independent experiment. *P* value was determined by two-way ANOVA. (**G**) Immunoblotting analysis of indicated proteins in HCT116 cells transduced with retroviruses encoding *PLEK2* shRNAs, YTHDF2 shRNAs or their combination. shNC represents a non-targeting shRNA. α-Tubllin was used as the loading control. (**H**) Quantitative PCR analysis of *TYMS* mRNA levels in HCT116 cells with *METTL14* knockdown upon the treatment of actinomycin D (10 μg/ml). Data were presented as mean±SD from three independent experiments. *P* value was determined by two-way ANOVA. shNC represents a non-targeting shRNA.


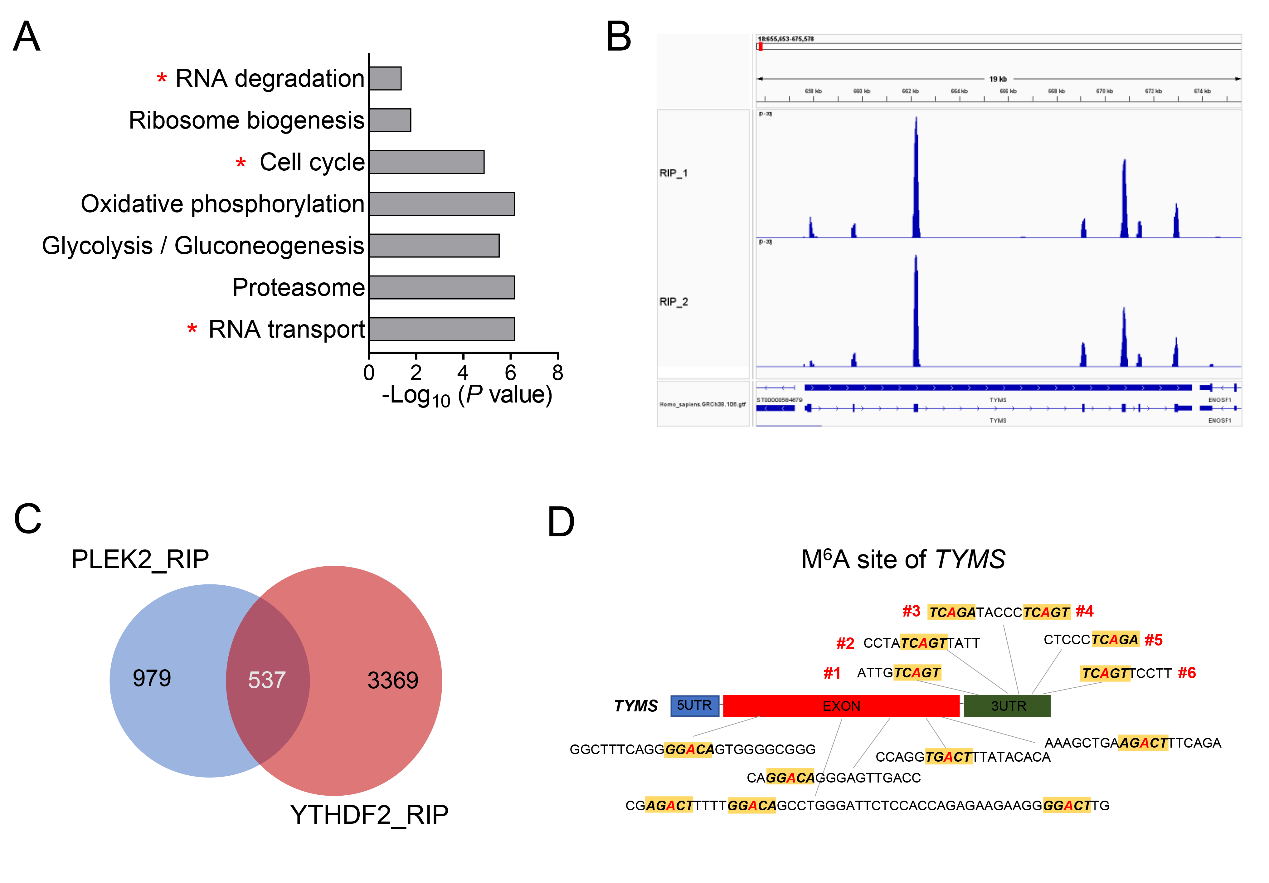


**Figure S6. PLEK2 and YTHDF2 cooperated to promote the *TYMS* mRNA stability in a m^6^A-dependent manner. Related to Fig. 5**

(**A**) Gene ontology enrichment of PLEK2-binding RNAs identified by RIP-Seq in HCT116 cells. (**B**) UCSC genome browser (http://genome.ucsc.edu/) of *TYMS* track peaks across the human genome (hg38 assembly) from PLEK2 RIP-Seq in HCT116 cells. (**C**) Overlap of peaks identified through YTHDF2-based PAR-CLIP (PMID: 24284625, YTHDF2_RIP) and the PLEK2 RIP-Seq peaks (PLEK2_RIP) in HCT116 cells. (**D**) High-confidence m^6^A sites of the *TYMS* transcripts was predicted from SRAMP (http://www.cuilab.cn/sramp) and published iCLIP binding data (PMID: 32492408).


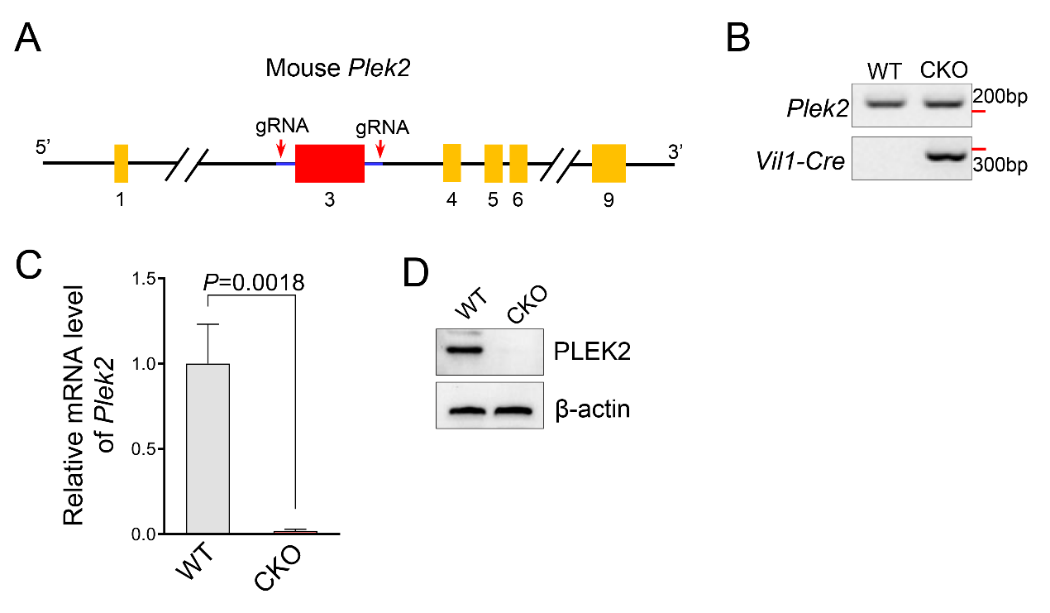


**Figure S7. Intestinal-specific knockout of *Plek2* mouse model. Related to Fig. 6**

(**A-B**) Schematic of *Plek2^fl^*^/fl^; *Vil1*-Cre gene-targeting strategy. (**C-D**) Quantitative PCR and western blot analysis of PLKE2 expression in intestinal epithelial cells from indicated mice. CKO: intestinal-specific knockout of *Plek2*.

**Supplemental Reference**

1 Le X, Mu J, Peng W, Tang J, Xiang Q, Tian S, et al.: DNA methylation downregulated zdhhc1 suppresses tumor growth by altering cellular metabolism and inducing oxidative/er stress-mediated apoptosis and pyroptosis. Theranostics 2020;10:9495-9511. DOI: 10.7150/thno.45631.

2 Chang F, Zhang Y, Mi J, Zhou Q, Bai F, Xu X, et al.: Rock inhibitor enhances the growth and migration of braf-mutant skin melanoma cells. Cancer Science 2018;109:3428-3437. DOI: 10.1111/cas.13786.

3 Qi J, Xu G, Wu X, Lu C, Shen Y, Zhao B: Peli1 and egfr cooperate to promote breast cancer metastasis. Oncogenesis 2023;12:9. DOI: 10.1038/s41389-023-00457-3.
